# Chronic Heart Failure and Exercise Intolerance: The Hemodynamic Paradox

**DOI:** 10.2174/157340308784245757

**Published:** 2008-05

**Authors:** Kent R Nilsson, Brian D Duscha, Patrick M Hranitzky, William E Kraus

**Affiliations:** Department of Medicine, Division of Cardiovascular Medicine, Duke University Medical Center, Durham, North Carolina, USA

**Keywords:** Chronic heart failure, exercise, skeletal muscle, gender, cardiac, resynchronization therapy (CRT)

## Abstract

Heart failure represents a major source of morbidity and mortality in industrialized nations. As the leading hospital discharge diagnosis in the United States in patients over the age of 65, it is also associated with substantial economic costs. While the acute symptoms of volume overload frequently precipitate inpatient admission, it is the symptoms of chronic heart failure, including fatigue, exercise intolerance and exertional dyspnea, that impact quality of life. Over the last two decades, research into the enzymatic, histologic and neurohumoral alterations seen with heart failure have revealed that hemodynamic derangements do not necessarily correlate with symptoms. This “hemodynamic paradox” is explained by alterations in the skeletal musculature that occur in response to hemodynamic derangements. Importantly, gender specific effects appear to modify both disease pathophysiology and response to therapy. The following review will discuss our current understanding of the systemic effects of heart failure before examining how exercise training and cardiac resynchronization therapy may impact disease course.

## INTRODUCTION

As the leading hospital discharge diagnosis for patients over the age of 65, chronic heart failure (CHF) represents a major source of morbidity and mortality in the United States[1]. Continued advances in the treatment of acute coronary syndromes promise only to increase the number beyond the over 550,000 new diagnoses made annually [2]. Despite advances in both pharmacologic and device therapies that have dramatically altered the natural history of CHF, disability associated with the symptom complex remains a major source of morbidity [2]. This symptom complex can be loosely divided into the acute symptoms of volume overload and the chronic symptoms of exercise intolerance, fatigue and exertional dyspnea. These chronic symptoms, in particular, dramatically impact quality of life.

Over the last two decades, several different lines of evidence have converged to identify skeletal muscle pathology as a major contributor to exercise intolerance and its attendant disability in chronic heart failure [3]. While numerous studies have linked enzymatic and histologic abnormalities with exercise intolerance, the underlying mechanisms driving these processes remain poorly understood [4-6]. Importantly, hemodynamic improvements do not acutely reverse this process. This “hemodynamic paradox” has spawned an enormous body of literature that collectively implicates dysfunction of oxygen utilization by skeletal musculature as being central to the symptom complex [7,8]. The following review will examine the role of the peripheral musculature in contributing to the morbidity associated with CHF before offering insight into how two seemingly disparate therapies – exercise training and cardiac resynchronization therapy – might improve exercise intolerance. In addition, we will explore how the various contributing factors are influenced by gender.

## HEMODYNAMICS

The reduced exercise tolerance observed in CHF has long been viewed as a direct consequence of decreased cardiac function. In support of this paradigm, exercise capacity as measured by peak oxygen consumption (VO_2_) is strongly correlated with cardiac output (CO). This correlation is supported by numerous studies over the past 15 years [9-11]. Resting indices of ventricular function (i.e., left ventricular ejection fraction, left ventricular end diastolic dimension, mean velocity of circumferential fiber shortening, and ratio of pre-ejection period to LV ejection time), however, are unrelated to exercise capacity or symptom status in CHF [9,12,13]. There are at least three reasons why resting indices of ventricular function do not relate to exercise capacity. First, resting parameters are unable to account for cardiac functional reserve. Second, resting indices do not address the role that both sympathetic drive and peripheral hemodynamics (i.e., the ability of the capillary bed to dilate or constrict in response to exercise) have on exercise capacity. Finally, resting indices of ventricular function fail to account for intrinsic alterations in the skeletal musculature that been found to at least partly account for the observed exercise intolerance in patients with CHF [5,14-16].

The importance of these last two points becomes apparent when one considers the determinants of peak oxygen consumption as described by the Fick equation, VO_2_=CO* ΔAVO_2_, where VO_2_ represents oxygen consumption, CO represents cardiac output, and ΔAVO_2_ represents arteriovenous oxygen difference. As a decrease in cardiac output is the *sine qua non* of chronic heart failure, augmentation of cardiac output would be expected to increase exercise capacity and peak oxygen consumption. However, in CHF, pharmacologic based improvements in cardiac output do not acutely translate into clinically significant improvements in either peak VO_2_ or symptoms [7, 8, 17]. In fact, it is as if the peripheral skeletal muscle abnormalities serve as a block in the translation of changes in cardiac output into improvements in exercise capacity and symptoms. Several different studies using vasodilators and inotropic agents have illustrated this point. In a study of ten patients with chronic heart failure hydralazine was shown to increase maximal exercise cardiac output (5.6 ± 0.7 to 6.7 ± 0.6 l/min; p<0.01) but had no effect on peak VO_2_ (787 ± 105 versus 779 ± 82 ml/min) [8]. Similarly, Maskin and colleagues evaluated the acute hemodynamic and metabolic effects of dobutamine in eight patients with NYHA III - IV chronic heart failure. While administration of dobutamine increased cardiac index during peak exercise from 2.67 ± 0.59 liters/min/m^2^ to 3.23 ± 0.78 liters/min/m^2^ (p<0.001), it did not increase exercise capacity (4.8 ± 1.5 min versus 4.5 ± 1.2 min) [7].

As VO_2_ is linearly related to cardiac output, the lack of improvement in VO_2_ with augmentation of cardiac output implies a corresponding reduction in ΔAVO_2_. Experimental models support this interpretation. If cardiac output is increased at rest and during exercise with dobutamine, there is almost no increase in oxygen consumption due to a proportionate decrease in ΔAVO_2_, as demonstrated in the aforementioned study of the hemodynamic affects of dobutamine on exercise capacity in severe CHF [7]. This “hemodynamic paradox” suggests that, in patients with CHF, alterations in peripheral oxygen consumption play a critical role in exercise capacity and fatigue. While these alterations do not directly affect the relationship between peak VO_2_ and cardiac output, they serve to inhibit the translation of acute changes in peak cardiac output to changes in peak VO_2_. 

## MORPHOLOGIC, HISTOLOGIC AND ENZYMATIC CHANGES OF SKELETAL MUSCLE IN CHRONIC HEART FAILURE

**Fig. (1):**
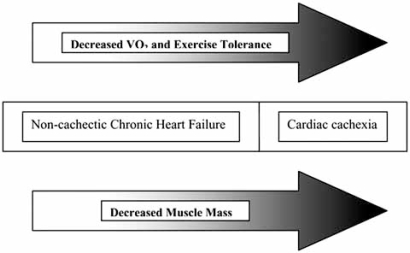
Relationship between exercise capacity and lean muscle mass [22].

Cardiac cachexia, defined as greater than a 6.0% weight loss over a six month period in the absence of other cachetic states (i.e., cancer), affects 10 - 16% of patients with chronic heart failure [18-20]. While cardiac cachexia represents the most extreme form of loss of muscle mass in chronic heart failure, a more subtle form of lean body mass changes clearly exists, as evident by the loss of skeletal muscle observed in patients with non-cachetic congestive heart failure (Fig. **1**) [21, 22]. Conceptually, the skeletal muscle alterations seen with CHF can be divided into histologic changes and biochemical changes.

Multiple different morphologic and histologic abnormalities have been described in patients with severe chronic heart failure. These include muscle fiber atrophy, altered capillary density, reduced maximal strength, and decreased electromyographic activity [21, 23-24]. Work by Sullivan and colleagues identified several additional histologic and morphologic changes that occur with chronic heart failure. Using vastus lateralis skeletal muscle biopsies from eleven patients with chronic heart failure and nine control subjects, Sullivan *et al*. demonstrated that patients with chronic heart failure have a reduced percentage of slow twitch type I fibers (36 ± 7% versus 52 ± 22%, p<0.05) and a higher percentage of type IIb fast twitch fibers (24 ± 9% *vs*. 11 ± 12%, p<0.05) [6]. Corollary studies exploring skeletal muscle myosin isoform expression in patients with CHF have demonstrated a reduction in myosin heavy chain type I. These results mirror the aforementioned histologic observations, as myosin heavy chain expression differs according to fiber type and is more abundant in type I aerobic fibers [16]. 

In addition to having an increased proportion of glycolytic fibers, patients with CHF also have clear alterations in their ability to supply oxygen to the muscle fibers *via* the capillary bed. Within a given individual, oxidative (red) skeletal muscles have a two- to four-times greater vascular density than glycolytic (white) muscles; cardiac muscle may be as much as ten times greater [25]. More important, this is a dynamic process, as evident by the increase in vascular density seen with exercise conditioning [26]. While capillary density has been variably identified as a histologic abnormality associated with chronic heart failure and exercise intolerance [27], recent studies that control for gender and exercise capacity (peak VO_2_) have demonstrated intriguing results. Duscha *et al.* compared 22 men with CHF to 10 sedentary controls matched for exercise capacity and found that capillary density as determined by endothelial cells per muscle fiber was decreased in men with CHF, implying that the abnormality was an intrinsic part of the disease state and not a consequence of deconditioning itself. Moreover, capillary density amongst men with CHF was inversely related to oxygen consumption (r = 0.479, p = 0.02) [15]. In their study of 38 patients with chronic heart failure (25 men, 13 women), Duscha and colleagues demonstrated that whereas men with CHF have an inverse relationship between capillary density and exercise capacity, no such relationship exists among women [28]. However, it should be noted that women with CHF had higher capillary density than their normal female counterparts, the opposite of the finding for men. 

Tyni-Lenne and colleagues have provided additional insight into the morphologic differences between men and women with heart failure. In contrast to men with heart failure, who have a decreased proportion of type I slow twitch fibers, women with heart failure have a normal proportion of type I fibers but decreased cross sectional area of both type I and type II fibers [29]. Two conclusions can be drawn from these studies: (1) the inverse relationship between capillary density and peak VO_2_ implicates an adaptive response in men to the disease state; (2) the myopathy associated with CHF is influenced by gender. Clearly, additional research is needed into how angiogenic factors control capillary density and how this is influenced by gender-specific effects. 

In addition to the histologic and morphologic changes seen in the musculature of patients with CHF, several studies have examined CHF-associated changes in the rate limiting steps of glycolytic and oxidative pathways [4,6]. Sullivan and colleagues explored this question by performing enzymatic analysis of muscle biopsies from 11 patients with CHF and comparing them to those of 9 control patients. Biochemical analysis demonstrated decreased activity of enzymes involved in oxidative pathways, such as succinate dehydrogenase (51 ± 15 versus 81 ±18 microM/g protein/min, p<0.001) and citrate synthetase (26 ± 7 versus 43 ± 20 microM/g protein/min, p < 0.05), but no change in the activity of enzymes involved in glycolytic pathways [4]. Sullivan’s work confirmed the results of Mancini and colleagues, who demonstrated a reduction in enzymes of aerobic metabolism such as beta-hydroxyacyl CoA dehydrogenase activity [23]. These enzymatic changes are mirrored histologically by a decrease in mitochondrial volume and functionally by a reduction in peak VO_2_. In their study of 57 patients with severe CHF, Drexler and colleagues found that both mitochondrial surface area and density decreased by approximately 20% when compared to controls. Interestingly, the reduction in mitochondrial volume was independent of patient age or etiology of heart failure [24].

**Fig. (2):**
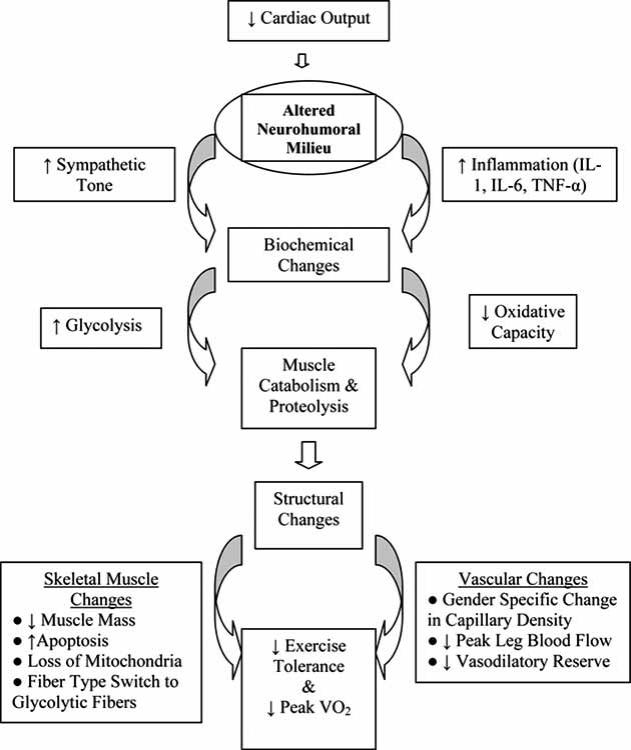
Proposed model of how a reduction in cardiac output leads to a series of downstream effects that ultimately limit aerobic metabolism in skeletal muscle.

Collectively, the morphologic, histologic and enzymatic changes seen in chronic heart failure are the manifestations of a systemic process where peak VO_2_ is limited not by hemodynamics, but by the oxidative capacity of the peripheral musculature. This observation has several implications. First, it raises the prospect that the myopathy of CHF is not being driven by hemodynamic derangements, but by alterations in the milieu of neurohormonal factors, metabolites and cytokines that bathe the skeletal muscle. Second, in order to reverse or prevent cardiac cachexia, these factors need to be removed or decreased. Finally, as the profound morphologic, histologic and enzymatic changes seen with CHF are reflective of changes in gene expression that have occurred over months to years, interventions designed to reverse the process must have an appropriate length of follow-up. Before discussing interventions, however, a discussion of the potential etiologic agents is warranted. While identified factors are certain to interact with one another, for simplicity they can be roughly divided up into immunologic and neurohormonal abnormalities. Fig. **2** provides a conceptual framework depicting how these factors might be interrelated. 

## IMMUNOLOGIC ALTERATIONS IN CHRONIC HEART FAILURE

Cytokines are known to play a direct role in promoting skeletal muscle wasting. Tumor necrosis factor-α (TNF-α), IL-6, and IL-1 have all been observed to be elevated in both humans and animal models of chronic heart failure (see Table **1**). TNF-α, in particular, appears to be central to this process, as it can directly induce skeletal muscle loss and is produced by the myocardium in response to increased ventricular wall stress [32-35]. Not only are serum levels elevated, but the magnitude of TNF-α elevation correlates with disease severity [36-37]. Despite its apparent central role in contributing to cardiac cachexia, therapeutic trials with anti-TNF therapy such as etanercept and infliximab have failed to show clinical benefit [36,38]. In fact, in the ATTACH trial, which randomized patients with NYHA Class III-IV chronic heart failure to placebo or infliximab, higher doses of TNF-α inhibition were associated with a three times increase in death or hospitalization at 28 weeks (hazard ratio 2.84, 95% CI 1.01-7.97, p=0.043) when compared with placebo [36]. Whether TNF-α inhibition reversed skeletal muscle myopathy was not evaluated and warrants further investigation.

The mechanism by which cytokines lead to muscle wasting may be related to inducible nitric oxide synthase (iNOS) induction [39]. Hambrecht and colleagues demonstrated that heart failure patients have increased expression of iNOS. In this same study, they demonstrated that this was inversely correlated with exercise capacity, mitochondrial oxidative capacity and mitochondrial structural integrity [40]. More recently, Adams and colleagues observed that increased iNOS levels correlated with increased skeletal muscle apoptosis [41].

## EFFECTS OF CHRONIC SYMPATHETIC ACTIVATION IN CHRONIC HEART FAILURE

The deleterious effects of chronic sympathetic activation in heart failure has been recognized for close to two decades, and its antagonism has resulted in a major advance in treatment, as evident by the clinical benefits of chronic beta blockade [42]. The role of chronic sympathetic stimulation in CHF associated skeletal muscle myopathy, however, has only recently begun to be appreciated. Chronic sympathetic activation appears to have at least three discernible effects on the skeletal musculature by: (1) impairing skeletal muscle blood flow; (2) up-regulating inflammatory cytokines; and (3) altering energy metabolism [31]. 

First, chronic sympathetic stimulation in patients with CHF promotes redistribution of blood flow to skeletal muscles through chronic vasoconstriction [43-46]. Chronic under perfusion of the capillary bed, in turn, promotes skeletal muscle ischemia, which leads to the generation of reactive oxygen species and muscle inflammation [18,48,49]. Second, a growing body of evidence suggests that sympathetic stimulation may be involved in the up-regulation of inflammatory cytokines. In patients with chronic heart failure, both TNF-α and Il-6 have been noted to increase in parallel with plasma catecholamines [50]. This association holds true in patients without heart failure, as sympathetic activation in humans competing in marathons has been associated with a 60 fold increase in cytokines, including IL-6 [31,51]. How sympathetic stimulation may lead to an increase in inflammatory cytokines is unclear, but may be related to the generation of reactive oxygen species and activation of NF-κβ [52]. Finally, chronic sympathetic stimulation *via* the beta adrenergic system alters the balance between glycogenolysis and gluconeogenesis, promoting a catabolic state. The resultant increase in glycogen phosphorylase activity has been implicated in the increased venous lactate levels seen in response to adrenergic stimulation [31, 53].

## THERAPEUTIC OPTIONS IN THE TREATMENT OF CHF ASSOCIATED MYOPATHY

As evident by the discussion above, the role of the peripheral musculature in the symptom complex associated with CHF is only beginning to be elucidated. While no therapy exists that directly reverses the myopathy, several different treatment strategies currently employed in the treatment of CHF have the benefit of improving exercise capacity, including exercise training and cardiac resynchronization therapy (CRT). Further research into the effects of these therapies on the skeletal muscle will hopefully further clarify the pathophysiology of CHF and lead to new treatment options.

## EXERCISE TRAINING IN CHRONIC HEART FAILURE

Several small studies have demonstrated a benefit of exercise training in terms of both peak oxygen consumption and other measures of exercise capacity [5,54-57]. The effects of exercise training on the skeletal musculature, however, have not been as well defined. While several studies have suggested that exercise training reverses CHF associated myopathy as evident by increases in fiber size, percentage of aerobic fibers, oxidative metabolism, capillarity and mitochondrial size, these results have not been reproduced in all studies (Table **2**) [15,30,58-65]. The discrepancy centers on whether exercise training reverses skeletal muscle myopathy or simply enhances glycolytic, but not oxidative, capacity.

Kiilavouri and colleagues randomized 27 patients with CHF to an aerobic training program (n=12) or control (n=15). The training group was subjected to a three month training program in which they exercised for 30 minutes, three times per week on a bicycle ergometer using a load that corresponded to 50 - 60% of their peak oxygen consumption. This was followed by a three month home training program. At the conclusion of the study, vastus lateralis biopsies were performed. While exercise capacity increased in the training group, enzymatic analysis of the muscle biopsies revealed an increase in activity of enzymes in the anaerobic glycolytic pathway. No increase activity was observed in the rate limiting enzymes in the citric acid cycle or in fatty acid oxidation [62]. This was reflected histologically, as training had no effect on the proportion of slow twitch or fast twitch fibers, nor did it significantly alter capillary density.

Additional studies have demonstrated that while exercise does not alter the proportion of slow twitch to fast twitch fibers, there is a trend towards an exercise associated* decrease* in the cross sectional area of type I slow twitch fibers (p=0.062) and an *increase* in the cross sectional area of type IIb glycolytic fibers (p=0.068) [63]. In contrast with these studies, a recent study by Keteyian and colleagues demonstrated an increase in the expression of the MHC I isoform, predominant in type I slow twitch fibers, in men with CHF who underwent exercise training [65]. Such changes were not observed in women, underscoring the importance of conducting further research into how gender influences both disease pathophysiology and treatment strategies [65]. It also points out the limitations of generalizing the results of small studies, and mandates additional large research trials that are adequately powered to discern differences amongst subgroups. 

In addition to affecting the morphologic, histologic and biochemical properties of skeletal muscle, exercise training has been shown to have an anti-inflammatory effect at the level of the skeletal muscle. In a trial of twenty men with stable chronic heart failure (EF 25% ± 2%) randomized to exercise training (n=10) or control (n=10), exercise training was shown to have no effect on *systemic* TNF-α, IL-1-beta, or IL-6 levels but to have a profound effect on *local* expression in skeletal muscle. In addition, exercise training reduced local iNOS expression by 52% (6.3 ± 1.2 to 3.0 ± 1.0 U, p=0.007) [30]. How exercise training alters the local expression of inflammatory cytokines is unclear, but may be related to the approximately 50% reduction in levels of serum catecholamines that accompanies exercise training in heart failure patients [5]. Finally, it is possible the exercise training might benefit individuals with heart failure by removing the aforementioned block between the conversion of changes in cardiac output through medical therapy to changes in exercise tolerance and peak performance.

While exercise training appears to improve the surrogate endpoints associated with CHF myopathy, the small sample size of published studies has precluded a determination of whether exercise training improves hard end points (i.e., mortality). The HF-ACTION trial is an ongoing randomized controlled trial studying the effects of exercise conditioning in over 2000 subjects with left ventricular ejection fractions of ≤ 35% and NYHA class II-IV heart failure. Primary outcomes include mortality and hospitalization for CHF. Importantly, individuals enrolled in HF-ACTION must be on a stable and optimal medical regimen for CHF that includes both an angiotensin converting enzyme inhibitor and a beta-adrenergic blocking agent. Hopefully, the results of HF-ACTION will address whether exercise training alters the natural history of CHF. Regardless of the outcome, HF-ACTION will provide insight into the mechanisms of exercise tolerance and will provide the backdrop for additional research projects.

## CARDIAC RESYNCHRONIZATION THERAPY AND EXERCISE CAPACITY

The advent of cardiac resynchronization therapy (CRT) for the treatment of patients with dyssynchrony and symptomatic CHF despite optimal medical therapy ushered in a new era in the management of CHF, one in which devices that optimize the performance of the heart complement pharmacologic based therapies. To date, several thousand patients have been included in CRT trials and collectively they have conclusively demonstrated that CRT is associated with improvements in both exercise tolerance and peak oxygen consumption [66]. Importantly, the follow-up in these trials was on the order of months, theoretically providing adequate time for the hemodynamic benefits of CRT to translate into alterations in the skeletal musculature (Fig. **2**). At the level of the heart, CRT leads to improvements in systolic (e.g., EF) and diastolic function, a decrease in mitral regurgitation, a reduction in LV size and a reduction in myocardial energy expenditure [67]. While the mechanisms by which CRT improves the symptoms of CHF has not been fully elucidated, the benefits appear to arise, at least in part, both directly from neurohumoral alterations and indirectly from exercise training. 

Several different studies looking at the effects of CRT on sympathetic stimulation have demonstrated, in aggregate, that CRT has a sympathoinhibitory effect [31]. While plasma catecholamine levels have yielded variable results in response to CRT, muscle sympathetic nerve activity studies have consistently demonstrated both acute and chronic reduction in sympathetic activity [31]. This is illustrated by Grassi and colleagues who conducted a study of 11 patients with EF<35%, QRS >130 msec and NYHA III-IV symptoms who underwent CRT. After ten weeks of CRT, microneurography demonstrated an approximate 30**% **reduction in sympathetic activity [68]. Theoretically, reduction in sympathetic activity would then set up a feedback mechanism that would reduce inflammatory mediators, promote more physical activity and decrease deconditioning, and increase exercise capacity, perhaps by preventing the vasoconstricting effects of circulating neurohumoral agents. To date, no study has examined the effects of CRT on skeletal muscle gene expression, biochemistry or histology. The ongoing GENSYNC trial will hopefully shed additional light on the mechanisms by which CRT affects the peripheral musculature by analyzing skeletal muscle biopsies prior to and six months after placement of a biventricular pacer.

## CONCLUSION 

Chronic heart failure imposes an incredible economic burden on society and is associated with significant morbidity and mortality. Although both the initial insult and etiology of chronic heart failure are germane to hemodynamics, measurements of central indices fail to fully explain the associated exercise intolerance as acutely normalizing these hemodynamic measures does not result in improved functional capacity. Instead, fatigue and exercise intolerance have been associated with immunologic, enzymatic and histologic changes in skeletal musculature. Therefore, the apparent “hemodynamic paradox” appears to be explained by peripheral maladaptations in skeletal musculature that limit oxygen consumption and the conversion of changes in cardiac output to changes in exercise tolerance. These associations are strongly influenced by gender and potentially reversed by a variety of non-pharmacologic treatments for CHF including exercise training and CRT. Additional research is needed to elucidate the molecular mechanisms of skeletal muscle dysfunction, to clarify how the phenotype is modified by gender, and to identify new therapeutic modalities.

## Figures and Tables

**Fig. (3). Potential Influence of Exercise Training in Patients with Chronic Heart Failure:**
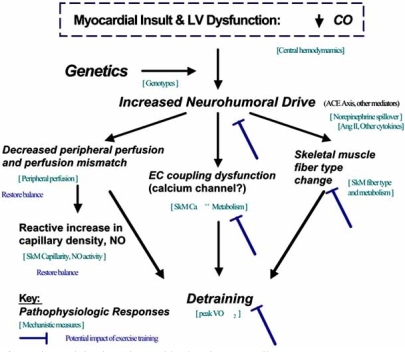
Fig. (**3**) represents a conceptual framework that depicts the interrelationship between hemodynamics, peripheral perfusion and skeletal muscle pathology in mediating the translation in cardiac output to impairment in function (peak VO2). In addition, the model depicts the potential role of exercise training in reversing this process.

**Fig. (4):**
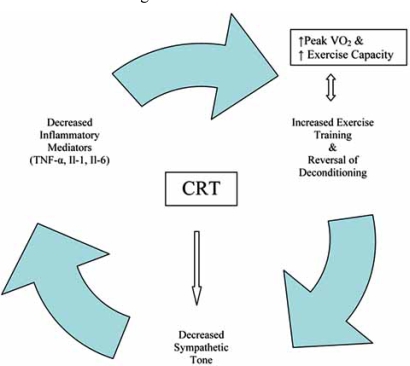
Proposed model by which Cardiac Resynchronization Therapy (CRT) may lead to a feedback mechanism that leads to improved peripheral oxygen utilization.

**Table 1:** Factors Involved in the Pathogenesis of Chronic Heart Failure Associated Skeletal Myopathy [30]

Factor	Effect	Effect of Exercise Training
TNF-α	Increased skeletal muscle apoptosis (Libera LD, J Mol Cell Cardiol 2001)	↓ skeletal muscle expression*
IL-6	Inversely correlated with muscle fiber diameter (Larsen AI, *et a.* Inter J Cardiology)	↓ skeletal muscle expression*
IL-1-beta	Inversely correlated with muscle fiber cross sectional area and IGF expression (Schulz pc *et a.*).	↓ skeletal muscle expression*
iNOS	Increased skeletal muscle apoptosis	↓ skeletal muscle expression*
Sympathetic Stimulation ^31^	Arteriolar Constriction and Decreased Capillary Blood Flow Increased Reactive Oxygen Species Apoptosis Inflammation and cytokine release Augmented glycogenolysis	↓ tonic sympathetic tone and sympathetic stimulation of muscle

**Table 2:** Effects of Exercise Training on Skeletal Muscle Histology, Enzymology and Immunology in Patients with Heart Failure

Study	Patient Population	Training Period	Findings
Hambrecht R, *et al.* (1995) [5]	22 males EF <40% NYHA II-III	6 months	19% ↑ in mitochondrial volume density 41% ↑ cytochrome c oxidase positive mitochondrial volume density.
Belardinelli R *et al.* (1995) [58]	23 men, 4 women EF 30% ± 5% NYHA II-III	8 weeks	24% ↑ in Type I and 17% ↑ in Type II cross sectional area 5% ↑ in capillary density 22% ↑ in mitochondrial volume density
Hambrecht R, *et al.* (1997) [59]	18 patients 26% ± 10%	6 months	41% ↑ surface density of mitochondrial cytochrome c 43% ↑ mitochondria cristae “Reshift” from Type II to Type I fiber type
Tyni-Lenne R, *et al.* (1997) [60]	16 women EF ~ 30% NYHA II-III	8 weeks training, 8 weeks non-training	32% ↑ in oxidative capacity
Tyni-Lenne R, *et al.* (1999) [29]	24 men and women EF 30% ± 11%	8 weeks of training	23% ↑ in oxidative capacity in patients undergoing cycle training 45% ↑ in oxidative capacity in patients undergoing aerobic knee extensor training
Tyni-Lenne R, *et al.* (1999) [61]	16 women EF 28% ± 8	8 weeks	Decreased number of Type I fibers Increased cross sectional area of muscle fibers
Kiilavuori K, *et al.* (2000) [62]	27 patients NYHA II-III	3 months	No change in capillary density No change in fiber type Increased anaerobic glycolysis No change in oxidative capacity
Larsen AI *et al.* (2002) [63]	15 men and women NYHA II-III EF 33% ± 5%	12 weeks	Non-statistically significant increase in IIb fiber type thickness Non-statistically significant decrease in Type I fiber type thickness
Santoro C, *et al.* (2002) [64]	6 patients	16 weeks	23.4% ↑ in mitochondrial size
Keteyian SJ, *et al.* (2003) [65]	10 men, 5 women NYHA II-III EF <35%	14-24 weeks	Increase in MHC class I in men but not women (38% v. -3%) No change in capillary density No change in enzymatic activity
Gielen S, *et al.* (2003) [30]	20 patients EF 25% ± 2%	6 months	No change in serum TNF-α, IL-1β, IL-6 Decreased local muscle TNF-α, IL-1β, IL-6
